# Impact of Managerial Reputation and Risk-Taking on Enterprise Innovation Investment From the Perspective of Social Capital: Evidence From China

**DOI:** 10.3389/fpsyg.2022.931227

**Published:** 2022-07-08

**Authors:** Shuang Wang, Shukuan Zhao, Dong Shao, Xueyuan Fan, Bochen Zhang

**Affiliations:** ^1^School of Business and Management, Jilin University, Changchun, China; ^2^Business School, Northeast Normal University, Changchun, China

**Keywords:** managerial reputation, risk-taking, innovation investment, corporate governance (CG), social capital theory, Heckman two-stage evaluation method

## Abstract

China’s enterprises established in the emerging economy are relatively short of technological innovation resources; therefore, these enterprises need to make use of managerial reputation to break through organizational boundaries in order to obtain richer social capital and reshape their technological creativity to cope with the complex and a changeable international economic situation. This corporate phenomenon also serves as the key for China’s economy to advance to the stage of high-quality development. Based on the panel data of Chinese A-share listed companies from 2007 to 2016, this paper adopts the Heckman two-stage evaluation model to empirically study the impact of managerial reputation on enterprise innovation activities, the moderating role of corporate governance, and the mediating role of risk taking. From the standpoint of social capital, the findings indicate that managerial reputation promotes enterprise innovation investment. The mechanism test reveals that this correlation is realized through the mediating role of risk taking. Furthermore, the promotion effect of management reputation on enterprise innovation investment is stronger when the enterprises adopt the CEO duality, the larger board size, higher management ownership, and stronger equity restriction. The conclusions of this study confirm the important role of social capital in enterprise innovation in the context of the Chinese economy. The study implications also enrich and expand the research on the influencing factors of enterprise innovation investment that focus on the managerial reputation and provide important business inspiration for enterprises to build reputation management strategy and promote the transformation and upgrading of local enterprises.

## Introduction

The Chinese economy is in a critical phase of transforming its development model, optimizing the economic structure, and augmenting the growth drivers. Enterprise innovation is the primary driving force that enhances the core competitiveness of the enterprises. It also promotes the upgrading of industrial structure and leads the macroeconomic transformation. At present, improving the independent innovation ability of local enterprises is the only way to realize the transformation of economic development from factor driven to innovation driven. Besides, it also helps to build a modernized economic system, and realize high-quality sustainable development goals (Wen et al., 2022a). Social capital is the sum of the network of relationships owned by individuals or social units. The actual and potential resources are obtained through and from the network of relationships (Bourdieu, 1986; Putnam, 1995). The social capital theory holds that enterprise innovation relies on the continuous input of resources in addition to general investment activities (Jefferson and Jian, 2006). Moreover, the input of social capital serves as a key part (Molina-Morales and Martinez-Fernandez, 2010; Perez-Luno et al., 2011). Consistent with this perspective, the existing literature majorly focuses on the social capital network constructed by enterprises and government institutions and investigates how to increase enterprises’ innovation resources and stimulate their innovation willingness (Kim and Zhang, 2016; Wrona and Sinzig, 2018; Amara and Khlif, 2020).

However, little attention has been paid to the social role of the manager, which is another important social capital of the firms for innovation activities (Wei et al., 2018). Managers’ social capital is a valuable asset that can generate positive cognition and judgment among the stakeholders (Kehoe et al., 2018). It also influences the availability and sustainability of enterprise innovation resources (Ranft et al., 2006). Besides, it enables the enterprises to yield sustainable profitability and deliver excellent financial performance (Li et al., 2020), thus influencing the investment behavior and innovation strategy (Bundy and Pfarrer, 2015). Therefore, it is of great theoretical value and practical significance to expand and deepen the research on enterprise innovation from the context of managers’ social capital as the main factor to improve the sustainable development of enterprises and promote the healthy and orderly operation of the social economy.

Managerial reputation refers to intangible assets acquired by managers through “public attention” and “emotional reactions of stakeholders” (Rindova et al., 2006). It represents a stable state generated by positive or negative media interactions, behaviors, and performances (Grigoriou and Rothaermel, 2014). It is a kind of social capital that highlights the enterprise’s ability to create value (Call et al., 2015). Managerial reputation can be developed and maintained through strategic actions (Long et al., 2015). There are different benefits for the enterprises in hiring managers with good reputations, such as improving the quality of corporate financial reports and financial performance (Bundy and Pfarrer, 2015) and motivating the managers to pay attention to the long-term performance fluctuations (Lindell and Perry, 2012). It also weakens the sense of risk aversion (Cho et al., 2016), improves the corporate image, and encourages the morale of employees and shareholders (Graham et al., 2005). Although there is little literature on the relationship between management reputation and innovation. Additionally, most of these studies are discussed from the perspective of mass communication theory instead of the social capital theory (Rindova et al., 2006). Since managerial social capital plays an imperative role in the enterprise innovation, therefore, it is important to explore whether managers’ reputations influence the firms’ risk-taking and innovation investment.

Based on the social capital theory, this study takes Chinese A-share-listed companies from 2007 to 2016 as a sample and adopts the Baidu index of managers’ positive media coverage to measure the managerial reputation and, afterward, discusses its impact on enterprises’ innovation investment. The results show that managerial reputation significantly promotes the firm innovation investment. Risk taking plays a mediating role in the relationship between managerial reputation and firm innovation investment. When the corporate governance model adopts CEO duality, the large board size, the higher proportion of management ownership, and the higher equity restriction ratio, the managerial reputation has a stronger promotion effect on the enterprise innovation investment. The further findings reveal that the managerial reputation of non-state-owned enterprises has a greater impact on enterprise innovation investment due to differences in the promotion mechanism and compensation system of the enterprises with different ownership. In addition, corporate social responsibility disclosure and philanthropy exhibit the same effect as individual managerial reputation on promoting enterprise innovation and development.

The theoretical marginal contribution of this research study has the following three aspects: Firstly, existing literature mainly studies the impact of social capital on enterprise innovation at the organizational level (Kim and Zhang, 2016; Wrona and Sinzig, 2018; Amara and Khlif, 2020). There is very limited literature that discusses the impact of management reputation on enterprise innovation, and most of these studies discuss the impact of “celebrity CEO” on enterprise innovation from the perspective of mass communication theory (Rindova et al., 2006). This research paper embarks on the managers’ deep strategic orientation instead of the “celebrities” or “stars” and other media to create a product surface phenomenon by examining the managerial reputation effect on enterprise innovation investment, expanding the research in the field of enterprise innovation, and establishing the reputation of the individual level for the management strategy of China’s listed companies in order to provide a new reference.

Secondly, scholars have also ignored the role of corporate governance mechanism in enterprise innovation and a lack of systematic and comprehensive analysis of each feature. This paper examines the internal mechanism and far-reaching influence of corporate governance structure on the relationship between managerial reputation and corporate innovation investment. The results show that the CEO duality, board size, proportion of management ownership, and equity restriction ratio create important boundary conditions and deepen the understanding and cognition of the mechanism of the corporate governance model in identifying the relationship between managerial reputation and enterprise innovation and development.

Thirdly, most of the previous studies focus on the direct impact of managers’ personal characteristics on enterprise innovation, and research in the field of risk taking is mainly concentrated in western countries. Besides, several variables, such as salary incentive (Cowen et al., 2016), corporate performance (Kim et al., 2015), organizational redundancy (Arrfelt et al., 2013), social-emotional wealth (Gomez-Mejia et al., 2018), managers’ cognition (Gamache et al., 2015), and psychological traits (Tang et al., 2015), are reported to influence the risk taking. However, research studies on the Chinese context are not only scarce but enterprise risk taking is also neglected as a mechanism test of the intermediate path. This study takes risk taking as an intermediate path to reveal its internal mechanism and propose potential solutions for risk decision-making in enterprise innovation management. Thus, it provides theoretical guidance on how to strategically use managerial reputation to build social capital and improve enterprise risk taking in the process of innovation.

## Theory and Hypotheses Development

Analysis of the R&D drivers remains the subject of economic research due to the importance of R&D investment in explaining economic growth. This study integrates the factors that can determine innovation input into the existing literature from three different theoretical structures: innovation leadership, management leverage, and business process. Firstly, the upper echelons theory holds that leadership at the individual level helps improve the effectiveness of interaction between team members (Sternberg et al., 2003). The leadership qualities, such as the education level, age (Mumford and Licuanan, 2004), values, experience, personality (Hambrick and Mason, 1984), professional knowledge, creative skills, and ability to process complex information (Mumford et al., 2002), are not only necessary for promoting innovation investment in the initial stage of setting innovation goals but also create conditions for the implementation of subsequent innovation practices. Secondly, dynamic capability theory believes that management leverage can improve firm innovation input at the organizational level. Managerial leaders implement deductive innovation strategies through direct leverage, decisions, and actions to achieve innovation input (Regnér, 2003). Similarly, managers employ indirect leadership skills to guide middle-management innovation teams to implement business process reengineering in order to improve innovation input (Jansen et al., 2009). Thirdly, typical process theory advocates that the core processes of innovation include initiation, portfolio management, development and implementation, project management, and commercialization. These business processes have an impact on innovation investment (Tsoukas, 1989; Van De Ven and Poole, 1995). However, few scholars discuss the impact of manager characteristics on firm innovation from the perspective of social capital theory.

The social capital theory is a favorable factor used by organizational researchers to elucidate the correlation between individuals, organizational networks, and the development of enterprises. Bourdieu (1986) claims that, since social networks are not innate, therefore, these networks must be constructed through investment strategies oriented toward the institutionalization of group relationships that can serve as a reliable source of other interests. Generally, the amount of social capital a person carries depends on the size of personal network connections on which that person relies as well as the amount and type of capital (e.g., economic or cultural) owned by each individual with whom that person is related. Coleman (1987) articulates the antecedents of possessing social capital (e.g., reciprocity expectations) and the consequences of having social capital (e.g., privileged access to information). Thus, it is crucial to differentiate between the type of capital itself and the ability to access it through the heterogeneity of organizational membership. This study groundbreakingly uses the number of positive news about managers in media reports to measure the managers’ reputation and explores the construction mechanism of managers’ reputation capital. Furthermore, it performs theoretical analysis and empirical tests on the mechanism and influencing factors of enterprise risk behavior and innovation strategy to fill the gaps in the existing literature. The study results reveal that the personal reputation strategy wins the goodwill of stakeholder groups, establishes a social capital network, and acquires the internal mechanism of scarce resources that are beneficial to enterprise development.

The role of managers in innovation activities and as the decision-makers in the enterprises has attracted the attention of academicians (Dai et al., 2021; Wen et al., 2022b). Managerial reputation has a vital role in influencing the initial perception and response of the evaluator. For internal stakeholders, personal reputation is a key factor that affects the managers’ risk appetite. It is also a source of innovational motivation and holds a strategic value for an enterprise (Pfarrer et al., 2010). For external stakeholders, amid lack of information and high uncertainty in the environment, a good managerial reputation builds the ex-ante trust of external stakeholders toward the enterprise. It not only declines the negative impact due to information asymmetry but also evades the impaired corporate financial performance as a guarantee of the innovation investment (Mishina et al., 2012).

Foremost, a good managerial reputation brings down the transaction and external supervision costs, as well as ensures a balanced enterprise innovation investment. The positive interaction between managerial reputation and other salient corporate characteristics or behaviors (e.g., production costs, product quality, and advertising investment) creates a virtuous cycle in which enterprises with good reputations have higher incentives to further enhance their reputations (Kehoe et al., 2018). Thus, the suppliers and buyers are less concerned regarding the contractual risks and quality risks when dealing with companies with a high managerial reputation (Li et al., 2020). Similarly, the consumers might pay a premium for the products provided by companies with a high managerial reputation to help these firms acquire various social capitals and uphold external innovation resources in the long run (Long et al., 2015).

Secondly, a higher managerial reputation can augment the investor confidence to invest in innovative projects and kindle the entrepreneurial drive for innovation. Managers with high reputations tend to highly recognize their R&D expertise and experience. These managers could avoid difficulties in innovation projects by precisely predicting the expected risks of R&D projects (Wei et al., 2018). Consequently, they tend to invest more in projects with higher risks, higher innovation, and higher challenges to match their excellent management levels and media evaluation in order to further consolidate their positions and reputation in the industry.

Thirdly, the managers’ reputation helps to decrease the enterprise losses by realizing the role of value maintenance in case negative events happen in the enterprise. In other words, when a negative event occurs, the reaction of external stakeholders starts from the general goodwill brought by the manager’s reputation. Furthermore, it is based on the objective assessment of the overall image of the enterprise. As a result, the manager’s reputation effectively decreases the adverse impact, outflow of social capital, and economic loss of the enterprise (Lindell and Perry, 2012). Parallel to this, positive comments from external stakeholders help companies attract investors’ attention and increase social capital and innovation investment. Therefore, this study postulates the following hypothesis:

Hypothesis 1: Managerial reputation promotes enterprise innovation investment.

Extant literature reveals that structural differences in CEO duality are reflected in the financial performance (Pathan and Faff, 2013), market valuations and stock returns (Erkens et al., 2012), compensation structure (Sierra et al., 2006), and risk-taking levels (Fortin et al., 2010). Finkelstein and D’Aveni (1994) believe that duality is a “double-edged sword” as there is an inherent balance between the unified command of the integration of the two functions and the independent supervision of the independent chairman of the board (Finkelstein and D’Aveni, 1994). According to the agency theory, the board of directors should be independent of management to prevent management from becoming entrenched (Eisenhardt, 1989). Therefore, CEO duality has a negative impact on corporate performance (Jensen, 1993). Contrarily, stewardship theory (Donaldson and Davis, 1991) and resource dependency theory (Boyd, 1995) argue that CEO duality promotes leadership unity and organizational effectiveness. Therefore, existing research studies on the relationship between the oneness of two roles and enterprise performance mainly rely on the two completely different theories called agency theory and stewardship theory. Boyd (1995) reports that dual roles provide unified command and decision-making speed in a highly uncertain environment; therefore, dual roles are beneficial for the enterprises. Peng et al. (2007) state that, since China is in a period of rapid institutional change and great environmental uncertainty, therefore, the potential benefits related to CEO duality proposed by management theory might exceed the potential agency costs emphasized by agency theory. However, a gap exists in the research on the boundary role of CEO duality in the correlation between managerial reputation and corporate innovation investment.

The corporate governance-related literature indicates that CEO duality is a vital tool for aligning the purpose functions of management and shareholders. It can enhance the corporate value by reducing the management conflict and augmenting the reputational recognition among the core stakeholders of the firm. Particularly, managerial reputation reflects the actual and observable economic behavior, as well as external perceptions of corporate behavior. The resulting advertising effect decreases the confusion and wait-and-see due to information asymmetry to investors (Castañer and Kavadis, 2013). Thus, the managers’ reputation in an enterprise with CEO duality can exert the greatest reputation effect, transmit high-quality corporate governance signals, absorb the scarce social capital of stakeholders, and ensure the smooth progress of innovation activities.

In contrast, the agency theory holds that CEO duality embeds managers with excessive discretion over shareholders wealth that leads the executive managers to make decisions to optimize their wealth or minimize their risk at the expense of shareholder value (Tuggle et al., 2010). Resultantly, the personal interests of general managers could influence the extent to which they participate in innovative projects (Yang and Zhao, 2014). Hence, consistent with the stewardship theory, CEO duality can be a tool to constrain management power, increase the influence of the general manager in decision-making, establish a reputation system for enterprise managers, attain more social capital, and increase enterprise innovation investment. Based on these arguments, the below hypothesis is proposed in this study:

Hypothesis 2: CEO duality positively moderates the correlation between managerial reputation and corporate innovation investment.

Existing studies have examined the effect of board size on corporate financial performance with mixed results. Some studies have shown that the board size has a negative impact on enterprise ROA, Tobin’s Q or ROE (Khan et al., 2015), which indicates a serious lack of coordination, flexibility, and communication among the board members. However, other studies have shown that board size has a positive impact on company performance. The board size reflects the trade-off between the cost and benefits of corporate supervision since a large-size board of directors promotes positive interactions between shareholders and other stakeholders (Arunruangsirilert and Chonglerttham, 2017). Meanwhile, board size has a significant impact on board independence and corporate performance (Rahman and Saima, 2018). In addition, scholars have made progress in the research direction of finding the optimal board size, believing that small or overcrowded boards destroy corporate value in different capacities. Particularly, Shawtari et al. (2016) report that the board size with the highest score of 4 has a positive and significant impact on performance. However, the positive effect of board size on corporate performance weakens when it is in the lower quintile of corporate performance, i.e., 10% (Shawtari et al., 2016).

Enterprises are relatively short of external resources due to the emerging status of the Chinese economy. As per the resource dependence theory, restriction of social capital scarcity on innovation activities determines the necessity of a large-scale board of directors. Firstly, large boards tend to enforce ethics, detect poor performance that affects managers’ reputations, correct problems such as managerial investment myopia (García-Sánchez et al., 2015), and tend to adopt good reputations in the decision-making of managers’ views on corporate sustainability, evading decisions that deviate markedly from the majority position, decreasing the variability of corporate innovation strategies, illustrating corporate investment value and innovation capabilities to stakeholders (Titova, 2016), and continuing the growth rate of corporate innovation investment. Secondly, large-size boards can provide firms with additional ways to connect with the external stakeholders to control the resources needed for firm innovation based on the resource dependency theory (Torchia and Calabro, 2016). Finally, large-size boards tend to include directors with diverse management and R&D experience. This diversity helps the board members to enhance the corporate social prestige and provide management with high-quality innovation strategies (Rahman and Saima, 2018). Thus, directors are motivated to uphold or build their reputation as supervisors through scale advantages, attract extra social capital, and increase investments in innovation practices. Based on these implications, the following hypothesis is also proposed in this study:

Hypothesis 3: The board size positively moderates the correlation between managerial reputation and corporate innovation investment.

Despite the growing research evidence that management with a higher shareholding exerts a significant impact on corporate financial performance, some studies claim that managers with stakes are more inclined to fulfill the expectations of powerful stakeholders; therefore, it exerts a positive impact on the correlation between managerial reputation and innovation (Jansen et al., 2006). Management with corporate stock dividends participates in the sharing of corporate value and acts in the interests of shareholders and other stakeholders. As a result, these managers are more inclined to monitor managers and build good personal reputations (Adams et al., 2005). Managers after holding corporate shares focus on the formulation and implementation of innovation strategies that are beneficial to the long-term benefits of the enterprise. Consequently, such managers promote the development of corporate innovation activities due to the dual dividends of personal benefits and organizational growth.

Although, other top management and board members are also involved in the strategic decision-making but the management holding corporate equity maintains a leading role in the strategy formulation by actively diverting the managers’ reputation toward the innovation strategies (Firth et al., 2006). Moreover, the major stakeholders often expect the management to become the main architect of the corporate innovation agenda through personal ownership, increasing corporate value and enhancing the stakeholders’ benefit. Thus, the management holding corporate shares tend to maintain a high reputation, win social capital for the enterprise, and safeguard the interests of different stakeholders through high-yield innovative projects (Crossland et al., 2014). Parallel to this, the below hypothesis is also presented in this study:

Hypothesis 4: Managerial ownership positively moderates the correlation between managerial reputation and corporate innovation investment.

Shareholders being the key stakeholders can use equity relationships to restructure assets, acquire knowledge, improve legitimacy, ensure the supply of innovation resources, and increase the security of new product markets (Stark and Vedres, 2006). The ownership structure represents the distribution of cash flow control and strategic decision-making rights among the shareholders (Block, 2012). Therefore, firms should recognize how the allocation of ownership structure affects the stakeholder expectations and innovation investment. The equity share not only gives minority shareholders the potential profit sharing and voting rights but also creates a feedback loop that ascertains the extent to which minority shareholders bear additional costs and other benefits (Huang and Zhu, 2015). In particular, a large number of small shareholders can coordinate and balance the interplay among all parties by actively supervising the operational processes, decreasing the likelihood of negative news, and ensuring the sustainability of innovation resources. Furthermore, minority shareholders influence the resource acquisition and knowledge creation ability of the enterprises through alliance network structure, promoting managers to build a reputation system, transmitting positive reputation signals to the potential investors, decreasing information asymmetry between shareholders and investors, and securing investment for innovation practices (Shapiro et al., 2015). Besides this, a balanced equity ratio restricts the encroachment of major shareholders’ interests, such as transferring the fixed assets, innovation output, and innovation resources of the business to another enterprise. It also prevents the major shareholders from benefiting at the expense of minority shareholders. Lastly, the equity restriction also reduces the resource acquisition, owing to managerial reputation, protects social capital, and ensures the sustainability of innovation investment (Borokhovich et al., 2006). Accordingly, this study proposes the following hypothesis:

Hypothesis 5: The equity restriction ratio positively moderates the correlation between managerial reputation and corporate innovation investment.

Numerous studies demonstrate that reputation is the perception of executives’ strategic decision-making and financial contributions, and the social capital it brings provides a resource guarantee for the enterprise innovation behavior (Fetscherin and Heinrich, 2015). Risk taking is a core component of strategic management research (Sitkin and Pablo, 1992; Bargeron et al., 2010). It represents the managers’ willingness to pursue high market returns and strategic management planning, and promotes the social and technological progress, such as short-term financial performance goals, long-term strategic flexibility goals, and social performance, by increasing R&D investment, promoting technological breakthroughs, and assuming the high risk of experimental failure, which can be used as a comprehensive indicator to measure the future development prospects of enterprises (Bargeron et al., 2010). In the corporate world, enterprises must inevitably face the uncertainty around the organization and take risks in order to improve the competitive advantage and strategic performance of the enterprises. Assuming the complexity between managerial reputation and firm innovation, it is essential to investigate the mediating mechanisms of risk taking. Therefore, this study focuses on corporate risk taking by reflecting a firm’s strategic choice of uncertain outcomes through the output of the organizational financial fluctuations rather than the risks faced by the organization in the future.

The managers with higher reputations tend to build a huge social capital network to access innovation resources since these managers exaggerate intrinsic aspirations and extrinsic expectations for constant excellence (Wade et al., 2006). The poor risk-taking ability of the enterprise might adversely influence the future salary premium and career prospects of the managers to a certain extent (Graffin et al., 2013). Managers protect their social status and reputation in the industry by managing their reputation capital to decrease the discrepancy between an individual’s social identity and external judgment, which, in turn, increases corporate risk taking. Besides, the rewards of managers’ reputations and the social-emotional connection through interactions with others create a commitment to their own reputation (Gomez-Mejia et al., 2018). Hence, the managers use identity control mechanisms to internalize reputation into identity, uphold a high reputation over time, and provide a “system of control” for subsequent risk perception and behavior (Helfat and Peteraf, 2015). The identity control mechanism guides the managers to establish positive interaction with stakeholders, attract the risk tendency, and cultivate risk-taking behavior of external investment. It also supports the managers to maintain substantial consistency among social reputation, external cognition, and behavior pattern by improving the enterprise’s risk-taking behavior, which reaffirms its high social status and good industry reputation.

Subsequently, enterprise risk taking determines the implementation of enterprise innovation strategies. Risk taking affects the decision-making of enterprises in multiple fields, including investment and financing decisions, management decisions, financial allocation decisions, talent introduction decisions, and R&D decisions (Hoskisson et al., 2017). The existing literature reports that firms with weak risk-taking ability pay less attention to R&D and innovation (Musteen et al., 2010). However, firms with strong risk-taking ability focus more on R&D (Barker and Mueller, 2002), pursue new technologies, new products, and new markets with high uncertainty (Li and Tang, 2010), and gain more innovation project investment (Tang et al., 2015). Risk taking improves enterprises’ awareness of innovative risk projects, capital accumulation and investment capacity, so it can attract more social capital to provide motivation and guarantee for innovative investment (Nadkarni and Chen, 2014).

Commonly, the managers’ reputation can bring capital accretion to enterprises, decrease the negative impact of enterprises, gain support from stakeholders, enhance the risk-taking ability, and, afterward, encourage the enterprises to formulate long-term strategies related to innovation, realize product updates, technology upgrades, and value chain creation. Therefore, this study believes that managers’ reputation promotes the enterprises to pay more attention to innovation activities and increase innovation investment through the enterprise risk-taking mechanism. Thus, the following last hypothesis is postulated in this study:

Hypothesis 6: Risk taking plays a mediating role in the correlation between managerial reputation and corporate innovation investment.

## Research Design

### Sample Selection and Data Sources

This study selects the panel data of Chinese A-share–listed companies from 2007 to 2016 as the research sample since the R&D data of the listed companies in China have been publicly disclosed since 2007, and the risk-taking method is projected on a 5-year moving window of a corporate-profit-margins basis. The sample selection for this research study is as follows: (i) excluded financial and insurance companies; (ii) excluded companies with special treatment or significant decline in their financial status under special government supervision (ST, *ST, special treatment), and the ratio of the annual decline ST company’s more serious special transfer company (PT, particular transfer); (iii) excluded companies with a consecutive reporting profit margin for < 5 years; (iv) excluded samples with missing data. Besides, this study also extracted the personal information of the board of directors, supervisory board, and executives of the listed companies from the China Stock Market and Accounting Research Database (CSMAR) database, and used Python software to crawl and manually search for missing data on Baidu webpages^1^ to collect, classify, and summarize the information. All research data are processed with STATA 16.0. The final study sample includes 21,352 valid observations of 3,089 listed companies.

### Dependent Variable

#### Innovation Investment

Innovation is one of the important tools for enterprises to increase their competitiveness and attain sustainable development. The International Economic Cooperation Organization (2012), used the R&D input intensity as the only indicator to measure the innovation investment (Chen et al., 2015). The R&D investment per $1,000 of sales is the specific measure of innovation investment (Betschinger, 2015). The *t* + 1 period of enterprise innovation investment (*RD_*t* + 1_*) is adopted to avoid the endogeneity problem and unbiasedly conduct this research.

### Independent Variables

#### Managerial Reputation

The managers’ reputation is considerably affected by media reports. Managers with better personal reputations and higher social praise tend to have a higher frequency of positive exposure in the news media as compared to ordinary managers. Therefore, this research study adopts the number of reports of the manager’s name in Baidu News as a proxy variable for the manager’s reputation. Additionally, the false exposure and negative news reports are filtered out that are promoted by the company’s own announcements or traffic advertisements. In particular, the number of citations is manually sorted out in various reports on the Baidu News webpage^1^ for “the name of the company’s management members and the company’s stock code where they currently work” from 2007 to 2016 in order to manually remove the negative and irrelevant news. The number of news reports is used to assess the managers’ reputations (Ye and Zhang, 2011).

### Mediating Variables

#### Risk Taking

Enterprise risk taking denotes a company’s willingness to take a higher risk of experimental failure by increasing R&D activities in order to promote technological breakthroughs for achieving short-term financial goals and long-term strategies. This enables the enterprise to chase high-profit returns, establish strategic management planning, and promote socio-technological progress. Flexible and social performance goals can be used as a comprehensive measure of future development prospects (Bromiley, 1991). As a sum of the measurement methods used in the existing literature, four methods are adopted to measure risk taking: tolerance of enterprises to experimental failure, the possibility of enterprise survival, enterprise R&D intensity and other policy behaviors, and enterprise performance (Bargeron et al., 2010). The assessment of risk taking based on the volatility of corporate earnings is more accurate and comprehensive as compared to the first three simple and intuitive measurement methods, since it is not easily affected by accounting measurement methods. Thus, aligned with the metric of John et al. (2008), this study uses the volatility of corporate returns to measure the level of corporate risk taking, which is divided into the following three steps. Lastly, the mediation effect is tested by Baron and Kenny’s (1986) step-by-step method.

1) Calculate the profit rate (the PF rate) of the company:


$${\mathrm{PFrate}}_{i,t}={\mathrm{EBIT}}_{i,t}/{\mathrm{Size}}_{i,t}$$

2) Calculate the industry-adjusted corporate profit margin (iR):


$${\mathrm{iR}}_{i,t}={\mathrm{PFrate}}_{i,t}-\frac{1}{N_{t,j}}\,\sum {\mathrm{PFrate}}_{i,t}$$

3) Calculate the standard deviation of the industry-adjusted 5-year moving window of corporate profit margins, that is, the risks taken by the company:


$$\mathrm{Risk}-{\mathrm{taking}}_{i,t}=\sqrt{\frac{1}{n-1}\,\sum {\left({\mathrm{iR}}_{i,t}-\frac{1}{n}\,{\mathrm{iR}}_{i,t}\right)}^{2}}$$

where *EBIT*_*i*,*t*_ denotes the profit before interest and tax of Enterprise *i* in Period *t*; *Size*_*i*,*t*_ denotes the total assets of Enterprise *i* at the end of Period *t*. *N*_*t*,*j*_ denotes Industry *j* in Period *t* number of enterprises; meanwhile, take *n* = 5.

### Moderating Variables

The heterogeneity of corporate governance plays a vital role in the correlation between managers’ reputations and corporate innovation investment. Thus, its boundary effect is also brought under analysis.

1) *CEO duality* (*duality*): When the chairman and the general manager of the company are the same person, take 1; otherwise, take 0 (Elsayed, 2010).
2) *Board size*. It was measured by the natural logarithm of the number of directors in the firm (Audra et al., 2007).
3) *Managerial ownership* (*ownership*): It is measured by dividing the number of shares held by management by the total number of shares (Luo, 2015).
4) *Equity restriction ratio* (*ERR*)*:* It is measured by the sum of the shareholding ratios of the second to fifth shareholders of the company divided by the shareholding ratio of the largest shareholder (Morck et al., 2000).

### Instrumental Variable

#### Investor Attention

In pursuit of their own economic interests, investors are indispensable scandal exposers, information catchers, followers of high-quality stocks, and forecasters of corporate development trends. Investors’ close attention to the behavior of enterprises and managers directly affects the managers’ reputation but exerts no direct impact on the innovation investment (Joe et al., 2009). Therefore, investor attention is added as an instrumental variable in the first stage of Heckman.

This research study directly assesses the investors’ limited attention through statistics of the Internet search index, contrary to the indirect measurement methods used in the previous empirical research methods. The Baidu search engine, developed by Baidu, is the most frequently used search engine by Chinese users (Bae and Wang, 2012). The Baidu Search Index is calculated as per the frequency with which Internet users search for keywords every day. In this study, using “enterprise stock name and stock code” as the keyword, the basic dataset is obtained by Web data crawling through Python software, and the information on the Baidu website is manually collected to supplement the database, and the daily Baidu search index is constructed, which is more intuitive. In addition, investor attention is also measured. The sample period is from December 31, 2007, to December 31, 2016. The logarithm of the Baidu index is also processed to avoid the problem of heteroscedasticity.

### Control Variables

Consistent with a large number of literature, this study also selects the following variables as control variables (McWilliams and Siegel, 2000; Mishina et al., 2012; Chen et al., 2015; Gomez-Mejia et al., 2018). We controlled for the following variables in terms of enterprise characteristics: *Firm Size*, represented by the natural logarithm of total assets. Enterprise size determines the innovation efficiency of a firm. Small-scale enterprises help to save management costs and use redundant resources for innovation activities; therefore, the prediction coefficient is negative. *Firm Age* is measured by the natural logarithm of the difference between 2021 and the year the firm was founded. The innovation consciousness and strategic flexibility of new innovative enterprises are strong, and the innovation resources accumulated by established enterprises are abundant. Therefore, the influence of enterprise age has two sides, and there is no need to predict the coefficient. The liability ratio (*LEV*) is estimated by total liabilities/total assets. Corporate debt has a crowding-out effect on innovation input; therefore our prediction coefficient is negative. Growth (*Growth*) is projected by dividing this year’s operating income/last year’s operating income – 1. Growth is of double significance to the innovation and development of enterprises; therefore, the coefficient is not predicted. Enterprise value (*TobinQ*) is measured by market value/book value. The greater the value of an enterprise is, the more it attracts the investment of external stakeholders in innovation projects; therefore, the prediction coefficient is positive. Profitability (*ROA*) is derived as net profit/total asset balance. The stronger the profitability of the enterprise, the more funds are available for innovation; hence, the prediction coefficient is positive. The cash flow ratio (*Cash flow*) is expressed as cash flow/total assets. The decline in cash flow motivates the companies to develop innovative strategies to maintain sustainable development, so our prediction coefficient is negative. Book-to-market (*BM*) can be expressed as book value/total market value. High book value reduces the innovation motivation; thus, the prediction coefficient is negative for this variable. The management expense ratio (*MER*) is represented as management expenses/operating income. The prediction coefficient is positive for MER since the higher the management costs, the higher the redundant resources. This indicates that the enterprise has enough capital to invest in innovation projects.

In terms of corporate governance, we control for the following variables: The proportion of independent directors (*Indep*) is represented by the number of independent directors/number of directors. Independent directors can supervise the utilization rate of innovation funds; therefore, our prediction coefficient is positive. The shareholding ratio of the largest shareholder (*LS*) is the number of shares held by the largest shareholder/total number of shares. The prediction coefficient is negative as the largest shareholder holds a large proportion, indicating a low degree of equity checks and balances, and a low degree of restriction on the opportunistic behavior of the largest shareholder, which will have a negative impact on innovation. *The institutional investor shareholding ratio* (*IIS*) is the total number of shares held by institutional investors/tradable share capital. The prediction coefficient should be positive as institutional investors play a supervisory role in the innovation activities of the enterprise. Major shareholder capital occupation (*MSCO*) is estimated as other accounts receivable/total assets. Other receivables have no impact on enterprise innovation; therefore, coefficient cannot be predicted in advance. For the big four accounting firms (*Big four*), it was 1; otherwise, it was 0. Working with the Big Four has a two-sided impact on innovation; therefore, predictions are not made.

For Year effect (*Industry*), 9-year dummy variables are set from 2007 to 2016. Twenty industry dummy variables are set for industry effect (*Year*) in accordance with the “2012 China Securities Industry Classification Guidelines”.

### Research Models

Models (1) and (2) are constructed to test Hypothesis 1. The dependent variable Mrep_*dum* in the first stage represents a continuous latent variable in the model (1). When the number of reports related to managers’ reputation is > 0, it is taken as 1; otherwise, 0. In addition, α_0_ denotes a constant term; β_1_ denotes the regression coefficient of the instrumental variable. *IA*_*i*,*t*_ represents the investor attention of Enterprise *i* in Year *t*. Similarly, *Control*_*i*,*t*_ stands for the control variable set of the equation. Lastly, Year is the year dummy variable, and Industry is the industry dummy variable with ε_*i*,*t*_ as the random disturbance term of Firm *i* in Year *t*.


$$\text{Mrep}\,\_\,{\mathrm{dum}}_{i,t}=\alpha_{0}+\beta_{1}\times {\text{Mrep}}_{i,t}+\beta_{2}\times {\text{IA}}_{i,t}+$$

(1) $$\Sigma \,{\text{Control}}_{i,t}+\text{Year}+\text{Industry}+\varepsilon_{i,t}$$

In Model (2), the second-stage dependent variable *R*_*Di*,*t* + 1_ indicates the innovation investment of Enterprise *i* in Period *t* + 1; α_0_ denotes a constant term. β_1_ is the regression coefficient of the independent variable, while IMR shows the inverse mills ratio calculated in the first stage. Furthermore, β_2_ denotes the regression coefficient of IMR, and Σ*Control*_*i*,*t*_ represents the control variable set of the equation. The Year is the year dummy variable, and Industry is the industry dummy variable, whereas ε_*i*,*t*_ is the random disturbance term of Enterprise *i* in Year *t*.


$${\text{RD}}_{i,t+1}=\alpha_{0}+\beta_{1}\times {\text{Mrep}}_{i,t}+\beta_{2}\times \text{IMR}+$$

(2) $$\Sigma \,{\text{Control}}_{i,t}+\text{Year}+\text{Industry}+\varepsilon_{i,t}$$

Model (3) is constructed to test Hypothesis 2, which adds the adjustment variable Duality_*i*,*t*_ based on the Model (2), as well as independent variables and adjustment of the interaction term of the variable. Model (3) focuses on the regression coefficient β_4_. If β_4_ is significantly greater than 0, the conclusion of Hypothesis 2 is verified, which indicates that CEO duality positively moderates the relationship between managerial reputation and firm innovation investment.


$${\text{RD}}_{i,t+1}=\alpha_{0}+\beta_{1}\times {\text{Mrep}}_{i,t}+\beta_{2}\times \text{IMR}+\beta_{3}\times$$

$${\text{Duality}}_{i,t}+\beta_{4}\times {\text{Mrep}}_{i,t}\times {\text{Duality}}_{i,t}+$$

(3) $$\Sigma \,{\text{Control}}_{i,t}+\text{Year}+\text{Industry}+\varepsilon_{i,t}$$

Model (4) is developed to test Hypothesis 3, which adds the adjustment variable *Boardsize*_*i*,*t*_ on the basis of Model (2), as well as independent variables and adjustment of the interaction term of the variable. It focuses on the regression coefficient β_4_. If β_4_ is significantly greater than 0, the conclusion of Hypothesis 3 is verified, which indicates that board size positively moderates the relationship between managerial reputation and firm innovation investment.


$${\text{RD}}_{i,t+1}=\alpha_{0}+\beta_{1}\times {\text{Mrep}}_{i,t}+\beta_{2}\times \text{IMR}+\beta_{3}\times$$

$$\mathrm{Board}\,{\mathrm{size}}_{i,t}+\beta_{4}\times {\text{Mrep}}_{i,t}\times \mathrm{Board}\,{\mathrm{size}}_{i,t}+$$

(4) $$\Sigma \,{\text{Control}}_{i,t}+\text{Year}+\text{Industry}+\varepsilon_{i,t}$$

Model (5) is constructed to test Hypothesis 4, which adds the adjustment variable Ownership_*i*,*t*_ based on Model (2), as well as independent variables and adjustment of the interaction term of the variable. Model (5) focuses on the regression coefficient β_4_. If β_4_ is significantly greater than 0, the conclusion of Hypothesis 4 is verified, indicating that management ownership positively moderates the relationship between manager reputation and firm innovation investment.


$${\text{RD}}_{i,t+1}=\alpha_{0}+\beta_{1}\times {\text{Mrep}}_{i,t}+\beta_{2}\times \text{IMR}+\beta_{3}\times$$

$${\text{Ownership}}_{i,t}+\beta_{4}\times {\text{Mrep}}_{i,t}\times {\text{Ownership}}_{i,t}+$$

(5) $$\Sigma \,{\text{Control}}_{i,t}+\text{Year}+\text{Industry}+\varepsilon_{i,t}$$

To test Hypothesis 5, Model (6) is constructed, which adds the adjustment variable ERR_*i*,*t*_ based on Model (2), as well as independent variables and adjustment of the interaction term of the variable. This model focuses on the regression coefficient β_4_. In case β_4_ is significantly greater than 0, the conclusion of Hypothesis 5 is verified, indicating that the equity restriction ratio positively moderates the relationship between manager reputation and firm innovation investment.


$${\text{RD}}_{i,t+1}=\alpha_{0}+\beta_{1}\times {\text{Mrep}}_{i,t}+\beta_{2}\times \text{IMR}+\beta_{3}\times$$

$${\text{ERR}}_{i,t}+\beta_{4}\times {\text{Mrep}}_{i,t}\times {\text{ERR}}_{i,t}+$$

(6) $$\Sigma \,{\text{Control}}_{i,t}+\text{Year}+\text{Industry}+\varepsilon_{i,t}$$

Model (7) examines the influence of risk taking (Risk−taking_*i*,*t*_) on the impact of managerial reputation (Mrep_*i*,*t*_) on innovation investment (RD_*i*,*t* + 1_) in Hypothesis 6. The mediating effect of β_2_ is the regression coefficient of the mediating variable. Model (7) focuses on the regression coefficient β_2_. If β_2_ is significantly greater than 0, it verifies Hypothesis 6 that managerial reputation promotes firm innovation investment through risk taking.


$${\text{RD}}_{i,t+1}=\alpha_{0}+\beta_{1}\times {\text{Mrep}}_{i,t}+\beta_{2}\times \mathrm{Risk}-{\mathrm{taking}}_{i,t}+$$

(7) $$\Sigma \,{\text{Control}}_{i,t}+\text{Year}+\text{Industry}+\varepsilon_{i,t}$$

### Heckman Two-Stage Method

There are different methods to solve endogeneity due to developments in microeconometrics. For instance, one-step/two-step system/difference GMM models are used to solve the problem of biased estimation caused by the influence of the dependent variable on past performance and omitted enterprise heterogeneity (Arellano and Bond, 1991; Blundell and Bond, 1998; Hillier et al., 2011). However, reverse causality tends to occur in this study when testing statistical relationships. This estimation bias, caused by sample self-selection bias, could lead to studies reaching flawed conclusions that could negatively impact managerial decisions. Although this research study has hypothesized the impact of managerial reputation on corporate innovation investment, perhaps, corporate innovation investment could, in turn, affect the strategic choices of corporate managers’ reputations. One of the main contributions of Heckman (1979) is to deal with the problem of sample self-selection bias. Thus, innovation investment in Period *t* + 1 is used as the dependent variable. Additionally, Heckman’s two-stage estimation method (1997) is adopted to handle the problem of sample self-selection bias due to reverse causality (Heckman, 1979) and make the result estimation more efficient.

The probability equation of managerial reputation is constructed in the first stage. The Probit model is used to estimate the “prechoice” of corporate managers’ reputations through a mixed sample of occurrence and nonoccurrence of explanatory variables. The dependent variable selects the dummy variable of the explanatory variable in the second stage and adds the instrument variable to obtain the IMR. Meanwhile, instrumental variables affect the probability of an observation appearing in the sample, correcting for potential endogeneity problems, but do not affect the final variables of interest in the second-stage ordinary least squares (OLS) model (Sartori, 2003).

In the second stage, this study constructs the regression equation of the influence of managers’ reputations on corporate innovation investment. Afterward, IMR is added to the second-stage equation as an error adjustment term, and instrumental variables are excluded. In case the IMR is significant, it is inferred that the sample has an estimation bias due to self-selection bias, and a treatment effect model has to be used to alleviate the estimation bias. The coefficients of the core explanatory variables are the results after considering the self-selection bias. If the IMR is not significant, it implies that the problem of sample self-selection bias is not obvious, and the model and results of the benchmark regression are reliable.

## Empirical Results and Analysis

### Descriptive Statistics and Correlation Matrix

Table 1 reports the observations, means, standard deviation, and minimum and maximum values of variables in the subject regression model and robustness tests. Of these, the total number of observations in the sample is 21,352. The average number of enterprise innovation investments in Period *t* + 1 is 4.52, and the maximum value is 15.6. This indicates that Chinese-listed companies attach great importance to innovation investment, laying a solid technical foundation for the sustainable development of enterprises. The average value of enterprise managerial reputation is 271.2, indicating that enterprises also attach great importance to building a good image through managerial reputation. The average value of CEO duality is 0.22, and the standard deviation is 0.42. This suggests that the corporate governance model of CEO duality has a small proportion in the listed companies. The maximum value of the managerial ownership is 0.61, and the average value is 0.11. This highlights that it has become a trend for companies to increase innovation investment through equity incentives. The average value of the equity restriction ratio is 0.65, and the standard deviation is 0.6, implying that most of the company’s ownership structure is relatively balanced, basically avoiding the situation of one dominant company. The average value of the nature of equity (SOE) is 0.43, and the standard deviation is 0.5. This shows that the sample has a large number of enterprises in China. The average value of investor attention is 6.1. This means that external investors pay close attention to the dynamics of the enterprise and exert a significant impact on the reputation of enterprise managers.

**TABLE 1:** Descriptive statistics.

Variables	Obs	Means	S.D.	Min	Max
RD*_t+1_*	21,352	4.52	5.48	0.00	15.6
Risk-taking	21,352	0.14	0.10	0.03	0.26
Mrep	21,352	271.2	584.6	0.00	17921
Mrep_dum	12,941	0.92	0.26	0.00	1.00
CSR	21,352	0.07	0.260	0.00	1.00
Phi	21,352	0.00	0.00	0.00	0.01
Duality	21,352	0.22	0.42	0.00	1.00
Board size	21,352	2.16	0.20	1.10	2.89
Ownership	21,352	0.11	0.20	0.00	0.61
ERR	21,352	0.65	0.60	0.00	0.99
SOE	21,352	0.43	0.50	0.00	1.00
IA	21,352	6.10	3.68	0.00	9.28
Firm size	21,352	21.9	1.29	15.58	28.51
Firm age	21,352	2.67	4.10	6.00	27.00
Indep	21,352	0.37	0.05	0.09	0.80
LEV	21,352	0.44	0.22	0.01	0.85
Growth	21,352	0.09	0.17	−0.39	0.30
TobinQ	12,941	2.25	0.72	0.85	5.23
ROA	21,352	0.04	0.06	−0.30	0.67
Cash flow	21,352	0.04	0.08	−1.94	0.88
LS	21,352	0.36	0.15	0.00	0.90
BM	21,352	0.88	0.98	0.00	13.71
IIS	21,352	0.36	0.24	0.00	1.87
MER	21,352	0.11	0.23	−0.01	16.61
MSCO	21,352	0.02	0.03	0.00	0.70
Big four	21,352	0.06	0.23	0.00	1.00

Table 2 shows that the results of the Pearson correlation coefficient matrix are all less than the critical value of 0.75, suggesting that the correlation coefficient between variables does not exceed the critical value, fulfilling the conditions for further research.

**TABLE 2:** A correlation matrix.

	1	2	3	4	5	6	7	8	9	10	11	12	13	14	15	16	17	18	19	20	21	22	23	24	25	26
1. RD*_t+1_*	1																									
2. Risk-taking	0.04***	1																								
3. CSR	0.03***	0.01*	1																							
4. Phi	0.00***	0.00**	0.00	1																						
5. Mrep	0.04***	0.00**	0.03	–0.00	1																					
6. LMrep	0.06***	0.00**	0.03	0.00	0.13***	1																				
7. Duality	0.10***	0.00	−0.03***	–0.00	0.03***	−0.13***	1																			
8. Board size	−0.09***	0.01**	0.05***	–0.00	0.02***	0.04***	−0.16***	1																		
9. Ownership	0.18***	–0.01	−0.04***	–0.00	0.06***	−0.28***	0.27***	−0.19***	1																	
10. ERR	0.16***	0.02***	−0.01**	0.00	0.07**	−0.12***	0.07***	0.00	0.29***	1																
11. SOE	−0.16***	0.00	0.06***	–0.00	−0.09***	0.17***	−0.27***	0.27***	−0.46***	−0.26***	1															
12. IA	0.08***	0.01	0.06***	0.00	0.12***	0.25***	−0.09***	0.12***	−0.26***	−0.15***	0.24***	1														
13. Firm size	−0.23***	−0.04***	0.14***	–0.00	0.18***	0.18***	−0.16***	0.26***	−0.29***	−0.14***	0.34***	0.52***	1													
14. Firm age	−0.11***	−0.051***	0.00	–0.00	0.00	0.25***	−0.09***	0.00	−0.26***	−0.05***	0.15***	0.23***	0.15***	1												
15. Indep	0.04***	−0.01**	0.00	0.00	0.00	–0.00	0.09***	−0.45***	0.09***	−0.01***	−0.07***	0.02***	0.03***	−0.01**	1											
16. LEV	−0.30***	−0.02***	0.05***	–0.00	0.04***	0.24***	−0.17***	0.17***	−0.37***	−0.18***	0.33***	0.33***	0.46***	0.21***	−0.02***	1										
17. Growth	−0.00*	–0.00	–0.00	0.00	0.00	0.00	–0.00	0.00	–0.00	–0.00	0.00	–0.01	–0.00	0.00	–0.00	0.00	1									
18. ROA	0.15***	–0.00	−0.02***	–0.00	0.00	0.04***	0.03***	−0.10***	–0.00	0.04***	−0.08***	0.04***	−0.26***	0.07***	0.03***	−0.08***	–0.00	1								
19. TobinQ	0.09***	0.02***	0.02***	0.00	0.06***	−0.22***	0.07***	0.00	0.20***	0.07***	−0.14***	−0.16***	−0.01*	−0.15***	−0.01**	−0.40***	0.00	–0.00	1							
20. Cash flow	–0.00	0.02***	0.02***	0.01	0.02***	0.03***	−0.02***	0.05***	−0.03***	−0.02***	0.04***	0.01	0.05***	–0.00	−0.03***	−0.12***	–0.00	−0.02***	0.33***	1						
21. LS	−0.14***	−0.01**	0.01***	–0.00	−0.07***	−0.05***	−0.04***	0.01**	−0.10***	−0.64***	0.19***	−0.06***	0.24***	−0.14***	0.04***	0.06***	0.00	−0.10***	0.10***	0.02***	1					
22. BM	−0.21***	−0.02***	0.08***	0.00	0.03***	0.14***	−0.13***	0.17***	−0.23***	−0.12***	0.30***	0.21***	0.60***	0.12***	0.02***	0.53***	0.00	−0.21***	−0.23***	−0.07***	0.11***	1				
23. IIs	−0.12***	−0.02***	0.09***	–0.00	0.07***	0.26***	−0.14***	0.14***	−0.37***	−0.12***	0.29***	0.14***	0.42***	0.15***	−0.01***	0.20***	0.00	0.02***	0.07***	0.11***	0.26***	0.18***	1			
24. MER	0.53***	0.00	−0.02***	–0.00	–0.01	0.01	0.02***	−0.06***	0.03***	0.06***	−0.08***	−0.01**	−0.15***	0.02***	0.02***	−0.09***	0.00	0.11***	−0.11***	−0.07***	−0.10***	−0.11***	−0.04***	1		
25. MSCO	−0.02***	0.03***	−0.01***	–0.00	0.01*	0.07***	−0.04***	−0.01**	−0.09***	−0.02***	0.00	0.08***	−0.01***	0.07***	0.01***	0.18***	0.00	0.05***	−0.15***	−0.10***	−0.08***	0.03***	−0.04***	0.07***	1	
26. Big four	−0.06***	–0.00	0.06***	–0.00	0.07***	0.02***	−0.07***	0.11***	−0.10***	−0.02***	0.14***	0.17***	0.38***	0.00	0.03***	0.10***	–0.00	−0.05***	0.02***	0.07***	0.14***	0.23***	0.18***	−0.03***	−0.05**	1

***, **, * represent the significant level of 1, 5, 10%, respectively.

The ordinary least square regression is used to avoid the multicollinearity between variables and obtain the moderate correlation between Variance Inflation Factor (VIFs) and independent variables. The results in Table 3 demonstrate that, among all variables, Firm size has the largest VIF, which is 3.26 and far lower than the threshold value of 10 (Cohen et al., 2003). Therefore, it is concluded that there is no multicollinearity problem in the results of this study.

**TABLE 3:** VIFs test results.

Variables	VIFs
Firm size	3.26
MSCO	2.57
ERR	2.33
IA	2.25
BM	2.19
LEV	2.15
LS	2.02
IIS	1.98
SOE	1.69
ROA	1.60
Board size	1.59
Indep	1.43
TobinQ	1.43
MER	1.28
Cashflow	1.24
Big four	1.24
Firm age	1.21
Mrep1	1.20
Duality	1.13
Ownership	1.07
Mrep_dum	1.06
CSR	1.02
Risk-Taking	1.02
Growth	1.01
Philanthropy	1.00
Mean VIF	1.63

### Regression Results

#### The Influence of Managerial Reputation on Enterprise Innovation Investment

Model 1 (Table 4) exhibits that the instrumental variables are significant (β = 0.1, *p* < 0.01), indicating that the basic model has a problem of sample self-selection, which had to be corrected by the Heckman two-stage regression method. Model 2 (Table 4) is used as the second stage basic model, and the IMR calculated by the first-stage model is added to revise the model. The regression results confirm that IMR is significant (β = –3.64, *p* < 0.01), and managerial reputation exerts a positive impact on corporate innovation investment (β = 0.27, *p* < 0.01). Hence, Hypothesis 1 is supported.

**TABLE 4:** Managerial reputation, corporate governance, and innovation investment usage.

Variables	First stage	Second stage				
	DV: Mrep_dum	DV: RD*_t+1_*				
	Model 1	Model 2	Model 3	Model 4	Model 5	Model 6
Firm size	−0.25***	0.00***	0.00***	0.00***	0.00***	0.00***
	–0.09	–0.01	–0.01	–0.01	–0.01	–0.01
Firm age	–0.22	0.00***	0.00***	0.00***	0.00***	0.00***
	–0.37	–0.04	–0.04	–0.04	–0.04	–0.04
Indep	–2.88	2.00**	1.80**	1.51	1.76**	2.03**
	–1.01	–0.22	–0.22	–0.22	–0.22	–0.22
LEV	−1.09*	−1.13***	−1.16***	−1.15***	−1.13***	−1.15***
	–0.57	–0.08	–0.08	–0.08	–0.00	–0.08
Growth	–0.06	–0.00	–0.00	–0.00	–0.00	–0.00
	–0.05	–0.00	–0.00	–0.00	–0.00	–0.00
ROA	5.68***	0.87***	0.76***	0.86***	0.86***	0.76***
	–1.09	–0.24	–0.24	–0.24	–0.74	–0.25
Cashflow	–2.04	−1.46**	−1.42**	−1.49**	−1.31*	−1.41**
	–0.72	–0.00	–0.00	–0.00	–0.00	–0.00
LS	−2.73***	−0.44***	−0.43***	−0.43***	−0.43***	−0.43***
	–0.38	–0.09	–0.09	–0.09	–0.09	–0.09
BM	0.301***	−0.17***	−0.17***	−0.17***	−0.17***	−0.17***
	–0.14	–0.16	–0.28	–0.11	–0.10	–0.16
IIS	1.01***	−0.56***	−0.22***	−0.53***	−0.63***	−0.23***
	–0.31	–0.06	–0.68	–0.08	–0.68	–0.08
MER	2.31***	−0.40***	−0.40***	−0.46***	−0.45***	−0.45***
	–0.56	–0.08	–0.08	–0.07	–0.08	–0.08
MSCO	3.71	−3.75***	−3.72***	−3.72***	−3.55***	−3.73***
	–2.83	–0.41	–0.41	–0.41	–0.41	–0.41
Big four	0.29	−0.11**	−0.14**	−0.12**	−0.14**	−0.14**
	–0.24	–0.05	–0.05	–0.05	–0.06	–0.06
IA	0.10***					
	–0.03					
IMR		−3.64***	−3.31***	−3.49***	−3.48***	−3.65***
		–0.36	–0.31	–0.36	–0.35	–0.35
Mrep_dum		0.98*	0.95*	0.93*	0.94*	0.97*
		–0.57	–0.57	–0.52	–0.56	–0.58
Mrep	0.02***	0.27***	0.23***	1.37***	0.23***	0.17**
	–0.45	–0.06	–0.06	–0.51	–0.07	–0.08
Duality			–0.17			
			–0.52			
Mrep*Duality			0.08***			
			–0.09			
Board size				−4.39***		
				–1.30		
Mrep*Board size				0.77***		
				–0.23		
Ownership					3.17***	
					–0.10	
Mrep*Ownership					0.33*	
					0.19	
ERR						–0.42
						–0.39
Mrep*ERR						0.11*
						–0.06
Constant	9.21***	6.72***	6.58***	16.06***	4.77***	6.92***
	–1.59	–1.24	–1.27	–3.04	–1.31	–1.31
Industry	Yes	Yes	Yes	Yes	Yes	Yes
Year	Yes	Yes	Yes	Yes	Yes	Yes
Observations	21,352	21,352	21,352	21,352	21,352	21,352

***, **, * represent the significant level of 1, 5, 10%, respectively. DV means dependent variable.

#### The Moderating Effect of Corporate Governance on the Correlation Between Corporate Managerial Reputation and Innovation Investment

Model 3 (Table 4) is used as the second-stage model. The IMR that is calculated by the first-stage model has been added to modify the model. The moderating variable CEO duality is added as the control variable, and the interaction term between the independent variable and the moderating variable is added to obtain a check for moderating effects. The results reveal that the IMR is significant (β = –3.31, *p* < 0.01), and the moderating effect is also significant (β = 0.23, *p* < 0.01). Thus, Hypothesis 2 is also supported, which states that CEO duality positively moderates the correlation between managerial reputation and firm innovation investment.

Model 4 (Table 4) demonstrates that the IMR is significant (β = –3.49, *p* < 0.01), and the moderating effect is also significant (β = 1.37, *p* < 0.01). Therefore, Hypothesis 3 is supported, that is, board size positively moderates the correlation between managerial reputation and firm innovation investment.

Model 5 (Table 4) displays that the IMR is significant (β = –3.48, *p* < 0.01), and the moderating effect is also significant (β = 0.23, *p* < 0.01). As a result, Hypothesis 4 is also supported, that is, managerial ownership positively moderates the correlation between managerial reputation and corporate innovation investment.

Model 6 (Table 4) indicates that the IMR is significant (β = –3.65, *p* < 0.01), as well as the moderating effect is also statistically significant (β = 0.17, *p* < 0.05). Thus, Hypothesis 5 of this study is supported, which holds that the equity restriction ratio positively moderates the correlation between managerial reputation and corporate innovation investment.

#### The Mediating Role of Risk Taking in the Correlation Between Managerial Reputation and Corporate Innovation Investment

Model 1 (Table 5) shows that managerial reputation promotes risk taking (β = 0.77, *p* < 0.01), and Model 3 (Table 5) also demonstrates that risk taking promotes corporate innovation investment (β = 0.9, *p* < 0.05). Thus, Hypothesis 6 is also supported, since risk taking plays a mediating role in the correlation between managerial reputation and corporate innovation investment.

**TABLE 5:** Managerial reputation, risk taking, and innovation investment.

Variables	DV: Risk-taking	DV: RD*_t+1_*	
	Model 1	Model 2	Model 3
Firm size	0.97***	0.01***	0.01***
	–0.28	–0.06	–0.06
Firm age	−0.11***	−1.17***	−1.10***
	–0.71	–0.15	–0.16
Indep	0.61**	0.08**	0.06**
	–2.94	–0.86	–0.82
LEV	−0.92**	−0.95***	−0.96***
	–1.22	–0.75	–0.76
Growth	0.41*	0.35**	0.29**
	0.00	0.57	0.61
ROA	0.72**	0.97***	0.89***
	–0.27	–1.98	–1.93
Cashflow	0.54***	−0.98***	−0.99***
	–0.36	–0.77	–0.67
LS	−0.66*	−0.11***	−0.15***
	–0.49	–0.46	–0.35
BM	0.29***	−0.05***	−0.03***
	–0.22	–0.06	–0.06
IIS	−0.42***	0.25***	0.24***
	–0.07	–0.04	–0.03
MER	0.57*	0.28***	0.26***
	–0.45	–0.24	–0.20
MSCO	0.36***	−0.08***	−0.13***
	–0.34	–0.91	–0.94
Big four	2.06**	0.17	0.12
	–0.10	–0.13	–0.14
Mrep	0.77***	0.06***	0.09***
	0.02	0.41	0.50
Risk-taking			0.90**
			–0.06
Constant	0.97***	0.44**	0.34**
	–0.77	–0.21	–0.19
Industry	Yes	Yes	Yes
Year	Yes	Yes	Yes
Observations	21,352	21,352	21,352
Adjusted R^2^	0.26	0.33	0.33

***, **, * represent the significant level of 1, 5, 10%, respectively. DV means dependent variable.

## Robustness Check and Further Exploration

### Robustness Check

**FIGURE 1:**
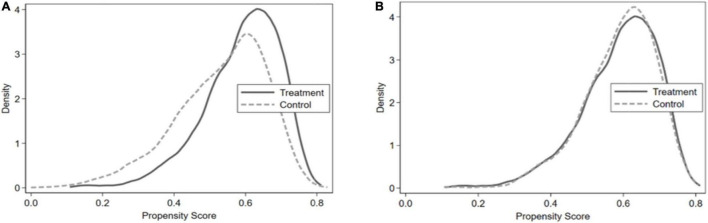
Probability distribution density function.

The following methods are used in this study to test the robustness of the regression results (Figure 1 and Tables 6–8).

**TABLE 6:** Robustness test: managerial reputation, corporate governance, and innovation investment.

Variables	First stage	Second stage				
	DV: Mrep_dum	DV: RD*_t+2_*				
	Model 1	Model 2	Model 3	Model 4	Model 5	Model 6
Firm size	0.19***	−0.33***	−0.29***	−0.28***	−0.28***	−0.25***
	–0.018	–0.09	–0.09	–0.09	–0.09	–0.09
Firm age	−0.051***	–0.29	–0.29	–0.26	–0.21	–0.22
	–0.144	–0.08	–0.08	–0.08	–0.05	–0.07
Indep	0.01	1.86**	1.68*	1.56	1.65*	1.90**
	–0.62	–0.80	–0.81	–0.85	–0.66	–0.87
LEV	−1.13***	−1.09*	−1.12**	−1.07*	−1.10*	−1.06*
	–0.08	–0.55	–0.54	–0.57	–0.56	–0.57
Growth	–0.00	–0.00	–0.00	–0.00	–0.00	–0.00
	0.00	–0.00	–0.00	–0.00	–0.00	–0.00
TobinQ	0.88***	5.38***	5.23***	5.43***	4.72***	5.42***
	–0.24	–1.09	–1.08	–1.09	–1.02	–1.09
Cashflow	–0.05	−1.29*	−1.26*	−1.25*	–1.11	−1.28*
	–0.16	–0.28	–0.27	–0.28	–0.29	–0.72
LS	−0.40***	−2.71***	−2.80***	−2.03***	−2.29***	−1.97***
	–0.09	–0.39	–0.38	–0.39	–0.36	–0.48
BM	−0.18***	0.32***	0.32***	0.31***	0.28**	0.30***
	–0.01	–0.11	–0.11	–0.11	–0.11	–0.11
IIS	−0.56***	1.03***	1.10***	1.03***	1.51***	0.96***
	–0.06	–0.31	–0.39	–0.39	–0.32	–0.31
MER	−0.44***	2.26***	2.20***	2.25***	2.06***	2.23***
	–0.08	–0.56	–0.56	–0.56	–0.55	–0.56
MSCO	−3.72***	3.50	3.57	3.43	3.04	3.30
	–0.41	–2.83	–2.83	–2.86	–2.81	–2.88
Big four	−0.14**	0.26	0.26	0.27	0.22	0.22
	–0.05	–0.24	–0.24	–0.25	–0.22	–0.24
IA	−0.10***					
	–0.03					
IMR		−3.65***	−3.61***	−3.64***	−3.31***	−3.49***
		–0.99	–0.94	–0.95	–0.91	–0.99
Mrep_dum		0.45***	0.42***	0.43***	0.42***	0.41***
		–0.13	–0.14	–0.18	–0.13	–0.13
LMrep	0.20***	0.16***	−0.16***	−0.14***	−0.19***	−0.16***
	–0.23	–0.05	–0.07	–0.07	–0.05	–0.05
Duality			0.05			
			–0.14			
LMrep*Duality			0.28***			
			–0.15			
Board size				–0.53		
				–0.33		
LMrep*Board size				0.17*		
				0.28		
Ownership					1.76***	
					–0.34	
LMrep*Ownership					1.49***	
					0.27	
ERR						0.11*
						–0.12
LMrep*ERR						0.24**
						–0.10
Constant	0.27***	0.82***	0.86***	0.81***	0.87***	0.99***
	–0.32	–1.51	–1.52	–1.65	–1.52	–1.52
Industry	Yes	Yes	Yes	Yes	Yes	Yes
Year	Yes	Yes	Yes	Yes	Yes	Yes
Observations	12,941	12,941	12,941	12,941	12,941	12,941

***, **, * represent the significant level of 1, 5, 10%, respectively. DV means dependent variable. LM rep means lag of Mrep.

**TABLE 7:** Robustness test: managerial reputation, risk taking, and innovation investment.

Variables	DV: Risk-taking	DV: RD*_*rm t* + 2_*	
	Model 1	Model 2	Model 3
Firm size	−0.01***	−0.01***	−0.08***
	–0.29	–0.07	–0.06
Firm age	−0.07***	−1.07***	−1.07***
	–0.71	–0.16	–0.14
Indep	−0.69**	0.99**	0.99**
	–0.92	–0.85	–0.88
LEV	0.92	−2.95***	−2.97***
	–1.22	–0.79	–0.76
Growth	−0.07*	0.26**	0.27**
	0.09	0.26	0.22
TobinQ	0.65	0.96***	0.89***
	–0.26	–1.98	–1.99
Cashflow	0.51***	−2.07***	−2.05***
	–2.36	–0.67	–0.68
LS	−2.44*	−2.98***	−2.97***
	–1.48	–0.42	–0.48
BM	0.30	−0.02***	−0.02***
	–0.22	–0.06	–0.01
IIS	−0.43***	0.25**	0.24**
	–1.06	–0.23	–0.22
MER	0.57***	0.26***	0.23***
	–0.45	–0.23	–0.27
MSCO	0.29***	−0.22***	−0.28***
	–0.35	–0.89	–0.88
Big four	2.06**	0.13***	0.11***
	–0.10	–0.13	–0.14
LMrep	0.13**	0.09***	0.09***
	–0.16	–0.03	–0.05
Risk-taking			0.03**
			–0.02
Constant	0.83***	5.35**	5.24**
	–6.73	–2.19	–2.18
Industry	Yes	Yes	Yes
Year	Yes	Yes	Yes
Observations	12,941	12,941	12,941
Adjusted R^2^	0.26	0.33	0.32

***, **, * represent the significant level of 1, 5, 10%, respectively.

**TABLE 8:** Equilibrium test results.

Variables	Mean		%reduct		*t*-test		V(T)/ V(C)
	Treated (Managerial reputation = 1)	Control (Managerial reputation = 0)	%bias	|bias|	*t*	*p* > |t|	
Firm size	22.14	22.12	2.100	89.50	1.030	0.305	1.090*
Firm age	2.727	2.722	1.400	83.90	0.700	0.484	1.100*
Indep	0.372	0.372	−0.800	92.10	−0.400	0.692	0.990
LEV	0.409	0.406	1.600	77.80	0.780	0.433	0.930*
Growth	0.284	0.234	0.300	87.80	0.810	0.417	5.800*
ROA	0.044	0.045	−1.000	25.30	−0.530	0.594	0.880*
Cashflow	0.045	0.045	0.000	85.80	0.020	0.983	0.940*
LS	0.351	0.350	0.500	62.30	0.260	0.793	1.050
BM	0.879	0.901	−2.400	84.90	−1.050	0.294	0.680*
IIS	0.392	0.385	3.000	57.80	1.510	0.130	1.000
Big four	0.054	0.050	1.600	71.50	0.800	0.421	0.922

***, **, * represent the significant level of 1, 5, 10%, respectively (Chang and Wu, 2020).

(1) In the robustness test, the natural logarithm of the number of positive citations of managers’ names in Baidu news reports as a surrogate variable for managers’ reputation is further evaluated, and the further tested results (Mrep_dum) are still robust.

(2) The number of news reports related to managers of listed companies in China and positive reports surged remarkably in 2012. For the listed companies, this is an important node for the managers’ reputation to absorb social capital and promote innovation and development. Consequently, the sample interval is shortened from 2007–2016 to 2012–2016, and the results are revalidated using the latest data, and the results are still robust.

(3) The abovementioned analysis used one period of lag in innovation investment as the dependent variable. But innovation activities have the characteristics of high risk and high uncertainty of returns. It usually took a longer period for the reputation of enterprise managers to influence the innovation activities. Hence, the lag period of the dependent variable is extended, and the *t*+ 1 period of enterprise innovation investment is replaced with the *t*+ 2 period of enterprise innovation investment, and the result is still stable.

(4) In the above analysis, the ratio of corporate net profit to total asset balance is used to measure corporate profitability (ROA). In the robustness test, profitability in the covariate is replaced with an enterprise value (Tobin’s Q), and the results are still robust.

(5) The propensity score matching method (PSM) is used to further examine the endogeneity problem. In specific operations, in order to improve the matching quality, only the individuals with overlapping propensity scores are usually retained, that is, if the propensity value of individuals in a treatment group is lower than the minimum value or higher than the maximum value of the propensity value of the control group, the individuals in the treatment group are removed. The putback match is allowed, that is, a successfully matched sample remains in the sample for other matches. Parallel matching is allowed, that is, if two values enter the matching range at the same time, the average of the sum of the two values is taken as the estimator. One-is-to-one scale K nearest the neighbor matching method in the caliper is selected. The results still remained robust. The results show that the two lines are more similar after matching, indicating that the experimental effect has been achieved (see Figure 1).

## Further Exploration

### The Moderating Effect of Equity Nature on the Correlation Between Managerial Reputation and Corporate Innovation Investment

The existing studies demonstrate that the heterogeneity of compensation systems and promotion channels leads to different innovation dynamics between state-owned enterprises (SOE) and non-state-owned enterprises (non-SOE) (He and Tian, 2013; Wei, 2021). State ownership and target setting: evidence from publicly listed companies in China. Nevertheless, the mechanism defining the role of corporate equity in the correlation between managers’ reputations and corporate innovation investment remains unclear. Therefore, we argue that the promotion effect of manager reputation on firm innovation investment is more significant in non-SOEs.

In this study, the SOE of the enterprise is determined based on the nature of the ultimate controller of the enterprise. In case the ultimate controller is an SOE, it is taken as 1; otherwise, it is taken as 0 (Guariglia and Liu, 2014). The below model is constructed to examine the moderating effect of the ownership nature of the firm on the correlation between managerial reputation and firm innovation investment:


$${\text{RD}}_{i,t+1}=\alpha_{0}+\beta_{1}\times {\text{Mrep}}_{i,t}+\beta_{2}\times \text{IMR}+\beta_{3}\times \text{SOE}+\beta_{4}$$

(8) $$\times {\text{Mrep}}_{i,t}\times \text{SOE}+\Sigma \,{\text{Control}}_{i,t}+\text{Year}+\text{Industry}+\varepsilon_{i,t}$$

Models 1 and 2 (Table 9) highlight that, compared within SOE, the managers’ reputations in non-SOE exert a more significant impact on innovation investment. Managers in non-SOE focus on personal reputation management, obtain more market legitimacy and innovation capital, perform higher-level innovation activities, and increase the level of innovation investment.

**TABLE 9:** Further analysis.

Variables						

	IV: Mrep		IV: Philanthropy		IV: CSR	
	DV: RD*_t+1_*		DV: Phi_dum	DV: RD*_t+1_*	DV: CSR	DV: RD*_t+1_*
	Second stage		First stage	Second stage	First stage	Second stage
	Model 1	Model 2	Model 3	Model 4	Model 5	Model 6
Firm size	0.19***	−0.28***	0.39***	0.32***	0.13***	0.35***
	–0.01	–0.09	–0.25	–0.97	–0.02	–0.08
Firm age	−0.78***	–0.26	0.02***	−1.22***	–0.02	−1.29***
	–0.04	–0.50	–0.04	–0.27	–0.05	–0.17
Indep	1.97**	1.81**	0.08***	2.42***	0.26	3.01***
	–0.22	–0.87	–0.29	–0.24	–0.33	–1.15
LEV	−1.13***	−1.11*	−0.32***	−3.14***	–0.09	−3.06***
	–0.08	–0.57	–0.12	–0.33	–0.13	–0.43
Growth	–0.00	–0.00	–0.15	−0.53**	−0.03**	−0.01**
	–0.00	–0.00	–0.01	–0.81	–0.06	–0.03
ROA	0.87***	5.32***	2.72***	0.72***	1.42***	0.22***
	–0.24	–1.10	–0.39	–1.94	–0.43	–1.45
Cashflow	−1.48**	−1.30*	0.21***	−1.24*	0.07	–1.25
	–0.16	–0.72	–0.27	–0.14	–0.30	–0.71
LS	−0.40***	−2.55***	−0.53**	−3.22***	−0.61***	−4.58***
	–0.09	–0.38	–0.12	–0.44	–0.14	–0.44
BM	−0.179***	0.321***	0.95***	0.87***	0.01	0.02
	–0.01	–0.11	–0.02	–0.66	–0.02	–0.42
IIS	−0.56***	1.09***	0.19**	0.43*	0.40***	1.13***
	–0.06	–0.31	–0.08	–0.23	–0.09	–0.31
MER	−0.45***	2.23***	−0.18**	2.57***	−0.71**	2.72***
	–0.08	–0.56	–0.25	–0.52	–0.35	–0.69
MSCO	−3.72***	3.12	−2.26***	−0.29**	–0.76	−4.79*
	–0.41	–2.83	–0.87	–2.69	–0.88	–2.81
Big four	−0.14**	0.26	0.16**	0.31**	0.04	0.43
	–0.05	–0.24	–0.06	–0.39	–0.08	–0.31
IA			0.01**		0.01***	
			–0.05		–0.04	
IMR	−3.61***	−3.48***		−3.37***		8.65***
	–0.36	–0.95		–0.99		–0.96
Mrep	1.00*					
	–0.56					
Mrep_dum		0.16***				
		–0.05				
Phi			0.15***	0.03***		
			–0.34	–0.08		
CSR						1.41***
						–1.95
SOE	0.26	0.18				
	–0.63	–0.13				
Mrep*SOE	−0.07**					
	–0.12					
Mrep_dum*SOE		−0.24**				
		–0.11				
Constant	0.99***	0.72***	−0.95***	−1.41***	−4.51***	−0.72***
	–1.52	–1.64	–0.44	–2.07	–0.49	–1.83
Industry	Yes	Yes	Yes	Yes	Yes	Yes
Year	Yes	Yes	Yes	Yes	Yes	Yes
Observations	21,352	21,352	21,352	21,352	21,352	21,352

***, **, * represent the significant level of 1, 5, 10%, respectively. Phi_dum means dummy variable of Phi.

### The Impact of Philanthropy on Innovation Investment

Godfrey (2005) reports that corporate philanthropy exerts a significant impact on shareholder wealth. Accordingly, it is inferred that philanthropy, as an organizational-level reputation management behavior, also exerts a crucial impact on innovation investment. The items marked as “donation” in the annual report of each company are adopted, the categories marked “fines” and other items explicitly not related to charitable giving are removed, and philanthropy (Phi) is measured by dividing the donation amount by the total assets (Ye and Zhang, 2011). The following model is constructed to examine the impact of corporate philanthropy on innovation investment:


$${\text{RD}}_{i,t+1}=\alpha_{0}+\beta_{1}\times {\text{Phi}}_{i,t}+\beta_{2}\times \text{IMR}+$$

(9) $$\Sigma \,{\text{Control}}_{i,t}+\text{Year}+\text{Industry}+\varepsilon_{i,t}$$

Models 3 and 4 (Table 9) show that corporate philanthropy increases the corporate innovation investment. Thus, philanthropy and managerial reputation are equally positive in promoting corporate innovation investment.

### The Impact of Corporate Social Responsibility Disclosure on Innovation Investment

McWilliams and Siegel (2000) claim that regardless of the heterogeneity of corporate social responsibility disclosure content, it is committed to decrease the negative externalities or generate positive externalities, and the results are beneficial to market or non-market stakeholders. Thus, it is further inferred that, when a corporate social responsibility (CSR) disclosure strategy is integrated into a corporate reputation strategy, one of its benefits is the potential to drive corporate innovation through both external and internal stakeholders, leading to increased corporate innovation investment. In addition, the data are obtained from the independent CSR disclosed by enterprises in the CSMAR database and examined whether to disclose the construction and improvement measures of the social responsibility system; 1 = disclosed, 0 = no disclosure (McWilliams and Siegel, 2000). To examine the impact of CSR disclosure on corporate innovation investment, the following model is constructed:


$${\text{RD}}_{i,t+1}=\alpha_{0}+\beta_{1}\times {\text{CSR}}_{i,t}+\beta_{2}\times \text{IMR}+$$

(10) $$\Sigma \,{\text{Control}}_{i,t}+\text{Year}+\text{Industry}+\varepsilon_{i,t}$$

Models 5 and 6 (Table 9) show that CSR disclosure promotes corporate innovation investment. Prominently, CSR disclosure helps managers focus on stakeholders who are less prominent but exert a significant impact on the company in terms of long-term financial performance, thereby focusing on increasing corporate integrity and augmenting corporate reputation.

## Discussion

Innovation is the primary driving force for enterprise development (Wen et al., 2022b). The increase of enterprise innovation investment is the primary factor to achieve high-quality development (Dai et al., 2021). More and more enterprises take social capital as the main source of innovation input to obtain external resources and promote innovation (Bundy and Pfarrer, 2015). However, the specific impact of corporate reputation capital on enterprise innovation investment is still in the black box. Therefore, the main purpose of this study is to empirically analyze the promotion effect of manager reputation on firm innovation investment and to investigate the moderating and mediating effects of corporate governance and risk taking between the relationship. In view of this, this paper uses the Heckman two-stage method to examine the impact of manager reputation on firm innovation investment and test its robustness and heterogeneity by taking Chinese A-share-listed companies as samples. The empirical results answer the above questions well.

First, the managerial reputation builds the trust of external stakeholders in the enterprise in advance (Kehoe et al., 2018), reduces the cost of external communication and supervision (Li et al., 2020), weakens the negative impact caused by information asymmetry (Wei et al., 2018), avoids the damage to the financial performance of the enterprise (Long et al., 2015), guarantees the enterprise to obtain and maintain the external investment in the long term, and provides a social capital guarantee for enterprise innovation investment (Lindell and Perry, 2012). This result not only provides empirical evidence for managers’ reputation capital to promote enterprises’ innovation behavior but also reveals the mechanism of action, which has an important contribution to the study of the relevant factors affecting enterprises’ innovation. Our results can help enterprises increase the proportion of highly reputational managers and obtain more social capital for enterprise innovation programs.

Second, in the boundary role of corporate governance, board size, managerial ownership, CEO duality, and equity restriction ratio positively moderate the correlation between managerial reputation and enterprise innovation investment. Specifically, the CEO duality makes the goals of management and shareholders converge, reduces the cost of communication and coordination (Pathan and Faff, 2013), increases the influence and efficiency of the general manager in decision-making (Castañer and Kavadis, 2013), and thus strengthens the contribution rate of the manager’s reputation in innovation input (Yang and Zhao, 2014). A large-scale board of directors can help mobilize the relationship network of directors (Khan et al., 2015), attract rich external resources (Shawtari et al., 2016), exert management and R&D functions (Titova, 2016), and guide reputable managers to invest in innovative projects that are beneficial to the long-term development of enterprises (Rahman and Saima, 2018). Managers with shares can adopt behaviors consistent with the interests of shareholders and other stakeholders, supervise managers to establish a good personal reputation (Adams et al., 2005), win social capital for enterprises, promote enterprise innovation strategy, and help stakeholders and managers to benefit themselves (Crossland et al., 2014). A balanced ownership structure can motivate minority shareholders to influence the resource acquisition ability of enterprises through alliance network structure (Block, 2012), motivate managers to build a reputation system, send positive reputation signals to stakeholders, reduce information asymmetry, and obtain innovative investment (Huang and Zhu, 2015). The results of this study expand the understanding of the boundary effect of corporate governance heterogeneity on innovation investment and contribute to the academic literature on corporate governance, which will guide enterprises to set up a scientific corporate governance model, promote the effect of managers’ reputation, attract social capital, and promote enterprise innovation.

Third, the results reveal that managers with a good reputation can bring multidimensional social capital to enterprises (Gomez-Mejia et al., 2018), guarantee the amount of capital reserves, and thus improve the risk taking capital of enterprises so that enterprises have sufficient redundant resources to invest in innovation projects (Helfat and Peteraf, 2015). Our findings expand the literature on risk taking and unpack the black box of risk taking in the relationship between social capital and firm innovation. Based on this finding, innovative enterprises should also increase the share of high-reputation managers, and build a competitive innovation system by improving the level of enterprise risk taking.

Moreover, we find that the promotion effect of manager reputation on firm innovation investment is more significant in non-SOEs. The reason is that the promotion of SOE managers is influenced by the tournament mechanism, which takes economic indicators, such as the growth rate of local GDP, as the evaluation standard, and it is not directly related to innovation performance (Boubakri et al., 2008; Lee et al., 2020). Thus, managers of SOE tend to give up opportunities to increase personal promotion capital through innovation and lack the motivation and pressure to build and sustain a reputation system (Baranchuk et al., 2014). In contrast, the compensation system of managers of non-SOE highly correlates with the corporate financial performance and innovation performance (Amore et al., 2013). Therefore, non-SOE managers must maximize personal and shareholder returns by augmenting personal reputation, building an innovation resource network, maintaining positive interactions with external investors, and completing longer-term and higher-level innovation tasks.

Finally, we find that enterprise philanthropy and CSR disclosure both promote the innovation investment. The reason is that corporate philanthropy and CSR disclosure both help to form positive moral capital, and provide insurance-like protection for relationship-based intangible assets and capital-based innovation activities. They play a positive role in corporate development by influencing stakeholder evaluations (Ye and Zhang, 2011). This invisibly drives the reputation management awareness of enterprises, decreases managers’ short-term behavior and opportunistic tendencies in investment toward the innovation projects, focuses on long-term decision-making such as innovation, and redesigns products, production processes, and productivity in the value chain, thereby promoting the intensity of enterprise innovation investment (McWilliams and Siegel, 2000). In the further exploration, we expand the research on reputation management at the enterprise level, and clarify the mechanism of the impact of corporate philanthropy and social responsibility disclosure on innovation. It provides business guidance for enterprises to develop individual and organizational reputation capital strategies.

## Conclusion

Based on the social capital theory, this study selects panel data of Chinese A-share-listed companies from 2007 to 2016 as research samples to examine the correlation between managers’ reputation, corporate governance, risk taking, and corporate innovation investment. In addition, it adopts the Heckman two-stage evaluation model to address the endogeneity problem of the sample selection bias. The research conclusions are as follows: Managerial reputation promotes enterprise innovation investment. In the boundary role of corporate governance, board size, managerial ownership, CEO duality, and equity restriction ratio positively moderate the correlation between managerial reputation and enterprise innovation investment. Moreover, managerial reputation primarily promotes enterprise innovation investment through risk taking. Furthermore, it is found that managerial reputation has a greater impact on innovation investment in non-SOEs. Additionally, corporate social responsibility disclosure and philanthropy attract social capital by increasing corporate reputation, which has the same effect on promoting enterprise innovation and development as individual managerial reputation.

The results of this paper have the following policy implications: Firstly, social capital serves as the key to China’s innovation-driven development strategy. Enterprises have long faced the problem of resource shortage in the process of China’s economic transformation, which has always been an unavoidable social problem in the history of China’s business development. The research conclusion of this study shows that the enterprise managers in the process of economic transformation should actively build a reputation system and stakeholders to form a virtuous cycle, reduce the external supervision cost, absorb the rich social resources, establish a positive corporate image, and improve the corporate ability to deal with the complicated financial activities. As a result, it will affect the risk-bearing level and innovative decision-making behavior of the enterprise. Secondly, the rational construction of corporate governance structure is crucial for listed companies due to the contribution of managers’ reputation capital in the enterprise innovation decisions. Therefore, enterprises should reasonably adopt the CEO duality model, expand the size of the board of directors, increase the proportion of management shares, and improve the degree of equity balance. Enterprises also need to make the interests of managers and shareholders converge, improve the efficiency of corporate strategic decision-making, reduce agency costs, fully mobilize the management talent and social network resources of directors and managers, and maximize the effectiveness and scientific nature of the corporate governance model in the enterprise innovation strategy. Finally, enterprises should make reasonable use of the resources brought by managerial reputation capital to improve their risk-taking ability, strengthen their awareness of innovative risk projects, improve the speed of capital accumulation and investment ability, promote the upgrading of industrial structure, lead the macroeconomic transformation, and improve the international core competitiveness of Chinese enterprises.

It is also to acknowledge that this study has several limitations. Firstly, although the study samples are widely representative and universal, there is no research on differences among different types of enterprises. In terms of sample selection, future research can pay more attention to the comparative analysis of enterprises of different types or regions, such as high-tech enterprises and non-high-tech enterprises, enterprises in less-developed regions and developed regions, and enterprises in the eastern, western, and central regions, etc. Secondly, although secondary data have the characteristics of high transparency and easy access, they do not have the characteristics of diversity and innovation and ignore the difference in data. In the follow-up research, it is suggested that the scholars can break through the research methods of secondary data and combine questionnaire surveys and interviews. Thirdly, there are still many aspects for investigating the boundary effect of corporate governance. Future research can start from the high-ladder theory and further investigate the characteristics of managers’ educational background, nationality background, gender, and other psychological characteristics, such as overconfidence or arrogance of managers.

## Data Availability Statement

The datasets presented in this study can be found in online repositories. The names of the repository/repositories and accession number(s) can be found below: China Stock Market and Accounting Research Database.

## Ethics Statement

Ethical review and approval was not required for the study on human participants in accordance with the local legislation and institutional requirements. Written informed consent from the patients/participants or patients/participants legal guardian/next of kin was not required to participate in this study in accordance with the national legislation and the institutional requirements.

## Author Contributions

SW and SZ contributed to the writing and formatting of the research. DS, BZ, and XF contributed to the editing and analysis of the research. All authors contributed to the article and approved the submitted version.

## Conflict of Interest

The authors declare that the research was conducted in the absence of any commercial or financial relationships that could be construed as a potential conflict of interest.

## Publisher’s Note

All claims expressed in this article are solely those of the authors and do not necessarily represent those of their affiliated organizations, or those of the publisher, the editors and the reviewers. Any product that may be evaluated in this article, or claim that may be made by its manufacturer, is not guaranteed or endorsed by the publisher.

## Footnotes

1
http://news.baidu.com/
